# Muscular Contraction—Cadaveric Rigidity—French and American Experiments, &c.

**Published:** 1852-02

**Authors:** 


### ANATOMY AND PHYSIOLOGY.

*Muscular Contraction—Cadaveric Rigidity—French and American Experiments, &c.*—It appears that the *Savans* of Paris have been very recently engaged in physiological experiments upon muscular contractility, and cadaveric rigidity, and they seem considerably elated, if not dazzled, by the new coruscations of light which these researches are supposed to shed in the realms of physiology. Whether this be, as it claims to be, a new burst of light, we will inquire into presently. In the meantime we propose to give a translation from a French Journal, narrating these discoveries, as reported by M. Leon Fourcault, on behalf of M. Brown-Sequard, as follows:

According to the opinion generally received, post mortem rigidity, which takes possession of the cadaver some time after the last breath, is wholly due to the mechanical effect of the coagulation of the blood in the animal tissues. The reasons given, have, at least, considerable plausibility. Among those individuals suddenly struck dead, whose blood preserves its natural elasticity, rigidity of the muscles manifests itself with great force, whilst it is scarcely seen in those who die after protracted diseases, or after copious hæmorrhages,* and still less among those who die asphyxiated by deleterious gases, the specific action of which prevents the coagulation of the blood. This contraction of the muscular system always disappears in advance of decided putrefaction, which destroys the organism, and subjects it to the reign of inanimate matter.

Hence we are readily induced to admit two kinds of death: the one, general, which supervenes first, at the moment when the heart ceases to beat, and which is in some sort only a displacement among the organic wheels of life, the intimate and harmonious action of which constitutes the unity of superior beings; the other variety of death supervenes subsequently in each individual wheel of the organism, as the consequence of the primary variety. Thus the general life may be abolished with the existence of the feeling or sentient unity, while, nevertheless, each organ preserves, during a time, a life of its own, after life proper, the persistence of which sustains itself above, and for some moments contends against the reign of inorganic matter. To this last phase of life cadaveric rigidity belongs, which, so far from being considered as the result of a purely mechanical action, bearing testimony to confirmed death, is, on the contrary, the last manifestation of muscular activity; at the moment of actual death, the muscular functions act for the last time, without guidance or aim, until the vital principle is completely exhausted.

The system of experiment to which M. Brown-Sequard boldly (*audacieusement*,) submits the living organism, seems to us to have been dictated by analogous considerations to those we have enumerated, and the results which he has obtained, favor the opinion that cadaveric rigidity is but life manifested in its utmost or last limits.

Having at first incidentally observed that the parts affected with this *post mortem* rigidity, (the assumed evidence of death,) may, under the influence of sanguineous injections, become again supple, and give signs of irritability, M. Brown-Sequard entertained this particular question, and, on varying his experiments, has arrived at the results which we are about to copy from him: In the dead bodies of rabbits and guinea-pigs, (which become rigid in from ten to twenty minutes,) he divided the aorta and cava, in the abdomen, just above the bifurcation of these vessels—that done, he put into the ends of these divided vessels quills or tubes of glass, and by means of these he connected the vessels of the dead with those of the living animals of the same species, so that the blood of the latter was injected into the arteries and veins of the former, thereby establishing the circulation in the inferior limbs of the dead. This transfusion resulted in the removal of cadaveric rigidity in from

*This opinion is erroneous. The most copious hæmorrhage in the subjects of yellow fever does not prevent cadaveric rigidity.

six to ten minutes, and two or three minutes later, the limbs responded by motions excited through mucular nerves.*

It is then proved by these experiments, that the nerves and muscles which have lost their excitability, may have the latter reproduced by the influence of the blood, and even for a quarter of an hour after *post mortem* rigidity had pervaded and ruled the muscles.

The same result was obtained by operating in another mode more simple and more easily repeated: A rabbit or guinea-pig was divided transversely on a level with the inferior borders of the kidneys—a ligature was applied on the aorta, which suppressed the vascular communication between the two sets of vessels; little by little the muscular activity declined in the inferior limbs, and was replaced, ordinarily, in less than half an hour, by rigidity. After having been abandoned to this state for fifteen or twenty minutes, the ligature was removed, whereupon the circulation was re-established, and then, as in the preceding case, rigidity was removed and excitability reappeared in the muscles and motor nerves.

Finally, in another series of experiments, M. Brown-Sequard investigated the voluntary motions and sensibility, with a view to ascertain whether they might not be re-established in limbs which, without having been separated from the nervous centres, could, nevertheless, be reduced to rigidity from the suspension of the circulation. With this view, he tied the aorta below the renal arteries, in vigorous rabbits; in less than ten minutes sensibility was abolished in the parts below, and in two minutes longer voluntary motion ceased; irritability still continued for nearly half an hour; afterwards rigidity took place, and was allowed to persist a quarter of an hour; at which time the ligature was removed, the circulation was restored, and, as had been expected, the blood brought back voluntary motion and sensibility.

These new researches bear out the following conclusions:

1st. That rigidity in the cadaver does not prove that the muscles are dead.

2d. That the motory and sensory nerves lose in the limbs all power to act where there is no circulation, but recover their functions from the action of the blood.

3d. That the limbs of mammiferous animals, after having been kept for fifteen or twenty minutes in a state of rigidity analogous to that in dead bodies, can be restored to their normal state, that is, to irritability, sensibility, and voluntary motion. LEON FOUCAULT."

The following account, translated by an individual unknown to us, is evidently a continuation of the experiments of M. Brown-Sequard, though the name is spelled differently. We correct the orthography in this particular.

"On the 18th of June, at eight o'clock in the morning, an assassin, condemned to death for murder, was executed at the Barrier St. Jacques. His headless body was given to a celebrated physiologist, M. Brown-Sequard, for the purpose of trying an experiment on the transfusion of

* The exciting agent used was, as usual, electricity.

blood. He had, in operations upon animals, noticed that the muscles which were just becoming rigid, seemed to resuscitate under the influence of fresh blood injected into the veins. The dead body preserved its muscular irritability until seven in the evening, when the stiffness, always consequent on death, seized upon the whole muscular system. As it was too late to obtain blood from the hospitals, and as that of animals seemed to promise but little success, M. Brown-Sequard caused an assistant to make an incision in his arm, from which he took about half a pound of blood. This was passed through a linen cloth, and injected into the radial artery of the subject, a little above the wrist. The corresponding vein was opened, and the natural blood of the dead man, now perfectly black from want of oxygen, was made to yield its place to the fresh blood injected. By continuing the injection, it passed through the capillary vessels, from the arteries to the veins, and flowed up through the orifice cut to allow the old blood to escape. Though it entered of a brilliant red color, it came out as black as the natural blood of the subject. But, being moved about in the air, it soon recovered its red hue, when it was again injected, to be still again disgorged by the open vein. In about half an hour the hand became sensitive, and moved convulsively under the discharge of an electric battery, which previously produced no effect. Out of the nineteen muscles of the hand, twelve recovered their natural irritability, or sensitiveness, and three of them contracted or expanded throughout their whole length. This state lasted from nine o'clock till midnight, when it began to yield to the rigidity natural to all bodies deprived of life. At six in the morning another experiment was tried, but neither the battery nor a fresh infusion of blood excited the least appearance of motion. The experiment seemed to decide, beyond a doubt, that in the human body, as well as in animals, the approach of rigidity may be deferred for a considerable period, by the injection of fresh blood, and that by the further application of electricity, the muscles may be made to move as in the living subject."

In another Journal we have met with a statement showing that the French *savans* regard artificial circulation as restoring to the dead both sensibility and motion, in part, at least. But what is the test used to prove "this high argument?" Electricity! "Sous l' influence de cette circulation et au bout d'une demi-heure, la main de supplicié, par une sorte de resurrection partielle, *redevint sensible et s'agita aux chocs reiteres des decharges electriques.* Cependant tous les muscles ne se montrerent pas egalement *sensibles.*" (See 2d. *L'Illustration, Journal Universel,* Aug. 14th, 1851.)

The fundamental propositions advanced in the above report, so far from being original and altogether new, are comparatively old. It is now a half a score of years since Dr. Bennet Dowler, of New Orleans, not only investigated experimentally, and preoccupied the grounds recently taken by the French *savans*, but a vast deal more, without the aid of electricity, (vulgarly called thunder and lightning.) He has not restricted his experiments to the inferior animals, as dead guinea-pigs and rabbits, (used by the French physiologists,) nor wholly to vivisections, but has experimented on hundreds of men, women, and children, without the all-powerful, but unphysiological forces generated by electrical batteries.

The general views of life and death presented in the above report, were long since brought forward in Dr. Dowler's essays on contractility, animal heat, capillary circulation, natural history of death, and, in other papers, with copious experimental illustrations. His ideas, and terms, (so far as the two languages will allow,) seem to have been adopted by MM. Foucault and Brown-Sequard, as novelties.

It is unnecessary to allude to Dr. Dowler's vast collection of unpublished researches upon these subjects. Soon after his experiments began, he published various extended monographs with experiments illustrative of the duration, degrees, progress, renewal, and decline of contractility and muscular motion, including the conditions that might be supposed to influence the muscles, as the nerves, the blood, hæmorrhages, the temperature, rigidity, diseases, and under the most varied manipulations and modifying circumstances. The reader and the French savans have only to look into the Medical Journals of Louisville, New York, New Orleans, and of other cities at home and abroad, to be convinced that the news from Paris is old, ("*trop tard*,") and that Dr. Dowler's claims to priority of discovery are entirely indisputable. It is now more than five years since Dr. Dowler, (as he informs us,) forwarded a number of copies of a pamphlet 39 pages, ("Experimental Researches on the post mortem Contractility of the Muscles,") to several members of the Academy of Medicine, and of the Academy of Sciences. Indeed, Dr. Dowler's discoveries in the muscular functions must have been known as early as the 5th of August, 1843. We have now before us a letter addressed to Dr. Dowler from the illustrious Louis, which we are permitted to use, and of which we will give a translation, showing that Dr. Dowler's paper entitled "Post mortem Researches," had been received in that city more than eight years ago. Now, in this very paper is announced Dr. Dowler's discovery of the excitation of post mortem contractility, by percussion, as well as several other discoveries, as post mortem caloricity, post mortem circulation, capillary action, etc., etc.

[TRANSLATION.]

*Sir and honored confrere:*—I know not how to thank you sufficiently for the extreme kindness with which you honor me, in addressing to me the two pamphlets which you have printed; one on pathological anatomy, and the other on what you call sun-stroke.

As to the subject of pathological anatomy, you have done, without doubt, that which no other physician has done till now; for it has been given to no person to make post mortem examinations a few minutes only after death, when cadaveric rigidity (one of the most certain signs of death,) exists, as yet to no great degree. You have been able to see that which few persons have seen—to establish lesions susceptible of changing quickly; and, if you have been able to collect in detail, and until the last hour of the patients, the symptoms which they have experienced, you will do a thing very useful to science to publish what you have seen.

As to what you call sun-stroke, the medical public cannot but read with a great deal of interest what you say, and, for my part, sir and honored confrere, I dare require you to pursue your researches, in order to have complete certainty that the facts observed by you, or rather the deaths of which you speak, are truly connected with the consequence of of the insolation.

Permit me, in conclusion, to persuade you to continue your valuable labors, to study rigorously the facts which you have collected, as you yourself propose. Have enough confidence in yourself to make them known to the medical public, to whom they will be serviceable, and please accept, sir and honored confrere, the assurance of my grateful and devoted sentiments. LOUIS.

*Paris, Aug.* 5, 1843.

Dr. Dowler's method (percussion) and results are alike original. Physiologists had used, and they still use electricity as the means of exciting muscular action! an agent of great power—one that produces irregular and convulsed motions in dead animals. Sir Charles Bell himself admits that results thus obtained are fallacious: "for the nerves, dead or alive, may convey the galvanic power, like a wet cord."*

It is unecessary to reiterate in this Journal the results of Dr. Dowler'sresearches upon these subjects. It is sufficient to say that he has shown that the muscular force may be repeatedly excited, exhausted, and regenerated for many hours, and even after the appearance of post mortem rigidity, without electricity, and after the removal of the nerves and blood. Cadaveric rigidity is often readily removed by art; and then, in many cases, muscular contractions will follow with perfect regularity, the cadaver raising his arm from the floor to his breast, carrying weights in his hand—after, as well as before the removal of the brain, cord, nerves, and blood.

The French savans have doubtless been deceived, as well in the nature as in the originality of their experiments. They had exhausted, temporarily, the muscular by the electrical force; they then set about injecting the blood-vessels with fresh living blood; in the meanwhile, the regeneration of the muscular force had been progressing, and had the operators injected no blood whatever, but delayed for an equal period, contractility would have returned; for the amputation of a limb, and the removal of the blood and blood-vessels, will not in the least diminish the contractility, though in the living state, Dr. Dowler has admitted that the blood and the nerves may contribute as auxiliary to the inherent forces of the muscles, being rather the essential conditions than the essential agents of motion, both voluntary and involuntary.

We venture to suggest another source of error into which the French *savans* have probably fallen. They tell us that cadaveric rigidity

*New Syst. 180.

having manifested itself in the guinea-pig, blood was thereupon injected, rigidity disappeared, and contractility returned. Now, we incline to think that it was the incidental manipulation of the animal, not the blood, that removed the rigidity; for Dr. Dowler's researches show, that in man, and in the alligator, how much soever they may be mutilated, rigidity may be, by forced motions, removed repeatedly, after which contraction can be excited for hours, and in the latter for three days! The rigidity will take place repeatedly, if the body be left perfectly undisturbed for a suitable time.

Dr. Dowler's researches prove that sensation and voluntary motion may exist independently of the brain. The French experimentalists seem to show that both of these fundamental functions can be revived, for a time, by the transfusion of blood from the living into the dead, even after the *rigor mortis*. We wait for further proof, as the subject is not sufficiently ripe for safe speculation.

Let the French *savans* look at page 54 of the July number of this Journal, where they will see in Dr. D.'s last contribution, a summary statement of the principal laws of muscular contractility, cadaveric rigidity, etc., deduced from experiments commenced eleven years ago.

With respect to this last contribution of Dr. D., (so fundamental, not to say revolutionary, in its bearings,) we may remark, that, judging from sundry letters and from notices of the medical press, so far as we have seen, its reception has been very flattering.

It has been urged as an objection to Dr. D.'s papers and essays, that they are not sufficiently practical in their spirit and bearing. To this we reply, that the writings of Hunter, Harvey, and some others, were looked upon by the less sagacious of their cotemporaries as of little practical utility at the time, and too speculative in their tone for the age; but the lapse of years and the researches of organic chemistry have established as indisputable truths many of these then supposed speculations, and mankind are at this moment receiving the benefits and blessings derived from the experiments of those illustrious minds. So, in like manner, we anticipate glorious results, at some future day, from the untiring labors and ingenious experiments of Dr. D.; and if we cannot apply to practical purposes—to the coining of money—the important discoveries in physiological sciences, which our confrere has from time to time published to the world, we indulge the pleasing hope that another generation will reap the full benefits of his labors, and render homage to his name.—*New Orleans Med. & Surg. Jour.*

